# Dissociation of Subjectively Reported and Behaviorally Indexed Mind Wandering by EEG Rhythmic Activity

**DOI:** 10.1371/journal.pone.0023124

**Published:** 2011-09-07

**Authors:** Jungang Qin, Christopher Perdoni, Bin He

**Affiliations:** Department of Biomedical Engineering, University of Minnesota, Minneapolis, Minnesota, United States of America; The University of Melbourne, Australia

## Abstract

Inattention to current activity is ubiquitous in everyday situations. Mind wandering is an example of such a state, and its related brain areas have been examined in the literature. However, there is no clear evidence regarding neural rhythmic activities linked to mind wandering. Using a vigilance task with thought sampling and electroencephalography recording, the current study simultaneously examined neural oscillatory activities related to subjectively reported and behaviorally indexed mind wandering. By implementing time-frequency analysis, we found that subjectively reported mind wandering, relative to behaviorally indexed, showed increased gamma band activity at bilateral frontal-central areas. By means of beamformer source imaging, we found subjectively reported mind wandering within the gamma band to be characterized by increased activation in bilateral frontal cortices, supplemental motor area, paracentral cortex and right inferior temporal cortex in comparison to behaviorally indexed mind wandering. These findings dissociate subjectively reported and behaviorally indexed mind wandering and suggest that a higher degree of executive control processes are engaged in subjectively reported mind wandering.

## Introduction

Selection, reduction of distracting information, and enhancement of performance are usually considered to be the characteristics of attention. Related mechanisms have been studied using various experimental paradigms and techniques [1]. Alternatively, the state of inattention to current activity, which is ubiquitous in everyday situations and impairs goal-directed behavior, has not received much attention. Mind wandering, as an example of such an inattentive state, refers to the situation in which we focus on thoughts and internal feelings that are unrelated to current activity [2]. Mind wandering is characterized by three features: Our attention becomes directed away from the performance of a primary task; our private thoughts and/or internal information become the focus of awareness; and we sometimes lack awareness that we are off task [3].

Neuroimaging studies have been conducted to uncover the neural correlates of mind wandering. For example, functional magnetic resonance imaging (fMRI) studies have demonstrated correlations between subjectively reported frequency of mind wandering and default network activation during low cognitive demand conditions [4], while individuals with higher propensities of mind wandering have been shown to activate stronger default network activities during highly practiced tasks when compared to novel tasks [5]. A recent fMRI study directly investigated brain activity linked to mind wandering using thought sampling and found that both the default network and executive brain system contribute to mind wandering [6]. Meanwhile, an event-related potential study, using the same paradigm as Christoff et al. [6], found the P300 component of external stimuli to be reduced prior to both the behavioral and subjective reports of mind wandering [7]. However, neural oscillatory activities related to mind wandering are still unclear, and, in particular, whether distinct rhythmic activities are linked to subjectively reported and behaviorally indexed mind wandering remains unknown.

To address these questions, we recorded subjects' electroencephalography (EEG) signals while asking them to perform a vigilance task with thought sampling, allowing for the collection of both subjective reports and behavioral indices of mind wandering. A previous simultaneous EEG and fMRI study of resting state found beta band power activity to be positively correlated with activity in areas belonging to the default mode network, whereas alpha band power activity was negatively correlated with activity in lateral frontal and parietal cortices that are known to subserve attentional processes [8]. An additional simultaneous EEG and fMRI study found EEG beta and gamma band power activities to be more correlated with identified positive fMRI resting state networks using independent component analysis than other bands' activities [9]. Theta band activity was also found under a mental mediation state [10], [11] and was correlated with default mode activity [12]. Thus, we systematically examined the pattern of neural oscillation activities within theta, alpha, beta, and gamma bands during both subjectively reported and behaviorally indexed mind wandering. In addition, a previous study using a similar paradigm found that the executive brain system contributes to mind wandering and more executive brain area activities were seemingly associated with subjectively reported rather than behaviorally indexed mind wandering [6]. Given that involvement of executive control processes were associated with enhanced gamma band activity [13], [14], we hypothesized that stronger gamma band activity would be observed during subjectively reported, rather than behaviorally indexed, mind wandering.

## Methods

### Subjects

Eighteen healthy undergraduate and graduate students (10 males and 8 females; mean age±SD = 22.83±3.35) participated in the current study. Four of the subjects (2 males and 2 females) were excluded from data analysis due to poor behavioral results or excessive artifacts during EEG recording. Thus, behavioral and EEG data were reported from 14 subjects (aged between 20 to 31 years; mean age±SD = 23.07±3.54). All were right-handed, had normal or corrected-to-normal vision, and were without neurological or psychiatric history. Prior to the study, subjects gave informed consent according to a protocol approved by the Institutional Review Board of the University of Minnesota.

### Task and procedure

Subjects were asked to perform a vigilance task with intermittent thought sampling probes. For the vigilance task, a single digit (0–9) was presented in the center of the screen every 333 ms. The target (whose presence required the pressing of a button) was the number 3 and appeared in 1.2% of trials. The low target frequency was intended to increase the incidence of mind wandering. The thought sampling probes were comprised of two questions regarding subjects' mental states immediately preceding the probe: Whether their attention was focused on the task or off the task; and whether they were aware of where their attention was focused. The answers to the questions were given on a 7-point Likert scale, ranging from “completely on task” to “completely off task” for the first question, and from “completely aware” to “completely unaware” for the second question. With this task, we were able to simultaneously examine subjectively reported and behaviorally indexed mind wandering; thought sampling probes provided subjective reports of mind wandering whereas performance errors in the vigilance task provided behavioral indices of mind wandering.

Similar to a previous study [6], in order to enhance the incidence of mind wandering and reduce learning effects during EEG recording, subjects completed three practice sessions before recording (each run lasting 14 min for a total of 42 min). During recording, subjects were required to complete eight task sessions. Each session consisted of 1764 trials (21 thought probes, 21 targets and 1722 non-targets). The order of events (targets and thought sampling probes) was pseudo-counterbalanced.

After EEG recording procedures, each subject was asked to fill out the daydreaming frequency and mind wandering subscales of the Imaginal Processes Inventory [15] to obtain measurements of individuals' propensities of mind wandering.

### Data recording

EEG recording was performed in a sound-attenuated and electrically shielded chamber. Continuous EEG was recorded using a Neuroscan® SynAmps amplifier, SCAN® version 4.2 software, and 62 scalp electrodes mounted on an elastic cap according to the extended 10–20 system. The grounding electrode for the active electrodes was placed anterior to Fz, while the reference electrode was located medially to Cz and CPz. Eye blinks and vertical eye movements were monitored with electrodes located above and below the left eye. Electrode impedance was kept below 5 kohms. The EEG signal was amplified (band pass 0.01–100 Hz) and digitized at a sampling rate of 1000 Hz.

### Data analysis

Off-line EEG processing was performed in EEGLAB [16]. The signal was first down-sampled to 250 Hz and average re-referenced. It was then epoched separately for each condition, beginning 4800 ms before the stimulus onset (thought probe or target onset) and continuing for 5600 ms (the time-window of interest was −4000 ms∼0 ms; additional time windows were added to eliminate boundary effects of time-frequency transformation). Trials contaminated by eye blinks, eye movements, or potentials exceeding ±200 µV at any electrode were excluded from further analysis. Due to differences in the number of trials between conditions (off-task/on-task; missed/hit target), we matched the trial number by randomly selecting trials from conditions with more trials to ensure that the number of trials was the same as the conditions with fewer trials.

The preprocessed EEG data were wavelet transformed using a complex Morlet wavelet from MATLAB Wavelet Toolbox with a center frequency of 1 and a bandwidth parameter of 2, with the frequencies of interest ranging from 3 to 70 Hz (theta (3–7 Hz), alpha (8–13 Hz), beta (13–30 Hz), and gamma (30–70 Hz)) with 1 Hz intervals between each frequency. The evoked spectral perturbation, a measure of the average power over epochs at given channel–frequency–time points, was then calculated to characterize rhythmic activities. The results were normalized by 1/*f* (*f* = frequency). These time-frequency analyses were conducted using ERPWAVELAB [17]. The statistical analysis of rhythmic activity differences between conditions (off-task vs. on-task; missed vs. hit target; subjectively reported vs. behaviorally indexed) was done using a nonparametric cluster-based randomization approach built into FieldTrip toolbox for EEG/MEG-analysis (FC Donders Centre for Cognitive Neuroimaging, Nijmegen, The Netherlands; see http://fieldtrip.fcdonders.nl/). This procedure defined clusters on the basis of the actual distributions of the data and tested the statistical significance of these clusters using a Monte-Carlo randomization method with correction for multiple comparisons [18], through which the clusters (several adjacent electrodes) that contained electrodes showing significant difference between conditions after multiple comparison correction could be obtained. The clustering used 500 randomizations and was performed in time (a total of 50 time points for a duration of 4 s) within each averaged frequency band (e.g. averaged 13–30 Hz for beta band). The t-statistic of paired t-tests was calculated on a cluster-level by taking the sum of the t-values within the respective cluster. A loose threshold was used (cluster survived a *p* value of 0.25) to ensure there was a positive/negative cluster in each condition. However, a more rigorous threshold was used (cluster survived a *p* value of 0.05) to investigate distinct neural activities between subjectively reported and behaviorally indexed mind wandering.

The linear constrained minimum variance beamformer implemented in Statistical Parametric Mapping 8 (SPM8) (Welcome Trust Center for Neuroimaging, London, UK, http://www.fil.ion.ucl.ac.uk/spm/software/spm8/) was used to perform the source imaging analysis of the EEG data within the time-frequency domain. We re-epoched preprocessed EEG data for each condition, beginning 4000 ms before stimulus onset and continuing for 4000 ms. Each subject's data were co-registered to the “standard” or “canonical” cortical mesh. EEG-BEM (Boundary Element Method) and standard models in SPM8 were selected as forward and inverse models, respectively. During beamformer analysis, the gridstep was set to 10 mm and regularization to 0. Due to the constraints of the analysis (i.e. the long time window present in the current study), we focused only on frequency bands with large ranges, such as beta and gamma. For each subject, we obtained the estimated source activities associated with subjectively reported mind wandering (off-task/on-task as active/baseline conditions) and behaviorally indexed mind wandering (missed/hit target as active/baseline conditions) within the beta and gamma bands. A one-sample t-test was then used to obtain group analysis (random effect analysis) results of neural activities linked to subjectively reported and behaviorally indexed mind wandering (multi-source activities). A loose threshold was used (voxels survived an uncorrected *p* value of 0.05, cluster size>100) to ensure there was activation under each condition. To investigate distinct neural activities between mind wandering and attention lapse, a group level two-sample t-test was conducted and a more rigorous threshold was used (voxels survived an uncorrected *p* value of 0.005, cluster size>10). This resulted in more focal brain areas due to the group random effect analysis.

## Results

### Behavioral results

**Figure 1:**
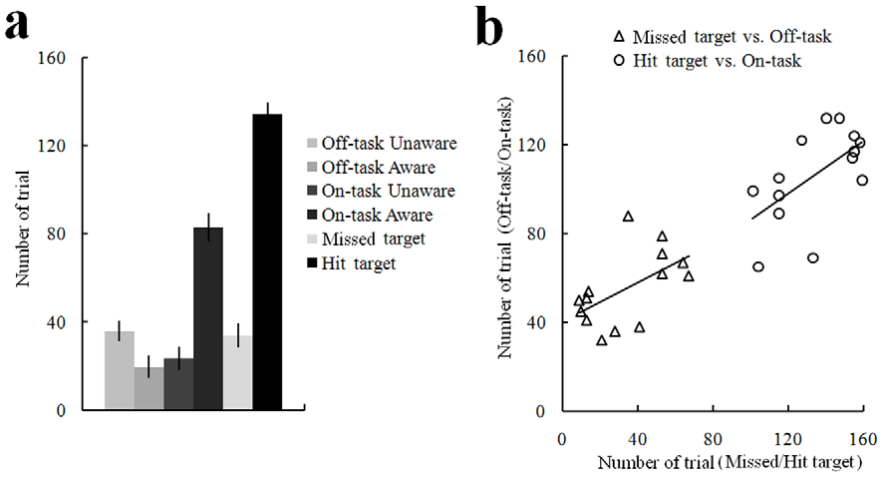
Behavioral results. (a) Trial number under each condition, error bars indicate standard errors of the mean; (b) Positive correlation between trial number of off-task and trial number of missed target, and positive correlation between trial number of on-task and trial number of hit target. The data of each subject is indicated by a triangle or circle. The lines represent the linear best fit.

The behavioral performances are shown in Figure 1a. After excluding the thought probes that were answered with the midpoint (4) of the 7-point scale, subjects reported being off-task in 32.95%±10.00% of the probes and on-task in 63.35%±12.59% of the probes. For the off-task intervals, subjects reported being unaware of where their attention was focused ‘on’ 66.28%±25.88% of the probes and aware of where it was focused ‘on’ 33.72%±25.88% of the probes. For the on-task intervals, subjects reported being aware of where their attention was focused ‘on’ 78.21%±17.98% of the probes and unaware of where it was focused ‘on’ 21.79%±17.98% of the probes. Repeated-measure analysis of variance on subjects' reports with Location of attention (on-task or off-task) and Awareness (aware or unaware) as independent within-subjects variables showed a reliable interaction effect (F(1,13) = 38.28, *p*<0.001). Post-hoc analysis found a significant effect of Awareness under on-task conditions (*t*(13) = 5.61; *p*<0.001) and an opposite marginal significant effect of Awareness under off-task conditions (*t*(13) = −1.857; *p* = 0.086), suggesting that subjects were more frequently aware of where their focus of attention was under on-task conditions, whereas they were less aware of where their focus of attention was under off-task conditions. Additionally, subjects missed 20.15%±12.58% of target trials during the vigilance task.

The relationship between behavioral performance and individual propensities of mind wandering (subjective rating results of daydreaming frequency and mind wandering subscales of the Imaginal Processes Inventory) were examined using correlation analysis (Fig. 1b). We found the number of off-task trials to be positively correlated with the number of missed target trials (*r* = 0.55; *p*<0.05) and the number of on-task trials to be positively correlated with the number of hit target trials (*r* = 0.60; *p*<0.05), suggesting a positive relationship between subjectively reported and behaviorally indexed mind wandering. In addition, rating results on daydreaming frequency were positively correlated with rating results on mind wandering (*r* = 0.84; *p*<0.001).

### Rhythmic activities related to subjectively reported and behaviorally indexed mind wandering

**Figure 2:**
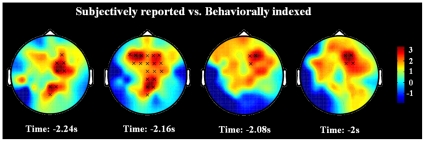
Topographical distributions of the enhanced gamma band activity associated with subjectively reported mind wandering relative to behavioral indexed mind wandering. The values are the statistical results of the differences. The ‘X’ marks the significant clusters of electrodes in the topographies.

To specify the rhythmic activities associated with subjectively reported mind wandering, we contrasted the rhythmic activities related to off-task trials with those linked to on-task trials. We failed to find any significant rhythmic activity associated with subjectively reported mind wandering under the acceptable threshold (cluster survived a *p* value of 0.05). Similar to subjectively reported mind wandering, we specified the rhythmic activities associated with behaviorally indexed mind wandering by contrasting the rhythmic activities linked to missed target trials with those related to hit target trials and did not observe any significant results. To test differences in the rhythmic activities between subjectively reported and behaviorally indexed mind wandering, we conducted further t-tests on the differentiated activities (off-task minus on-task; missed target minus hit target). Interestingly, we found that subjectively reported mind wandering evoked more gamma band activity over bilateral frontal-central areas than behaviorally indexed mind wandering (Fig. 2; Cluster *p* value = 0.04; time from −2.24 s to −2 s).

### Source imaging using the linear constrained minimum variance beamformer

**Figure 3:**
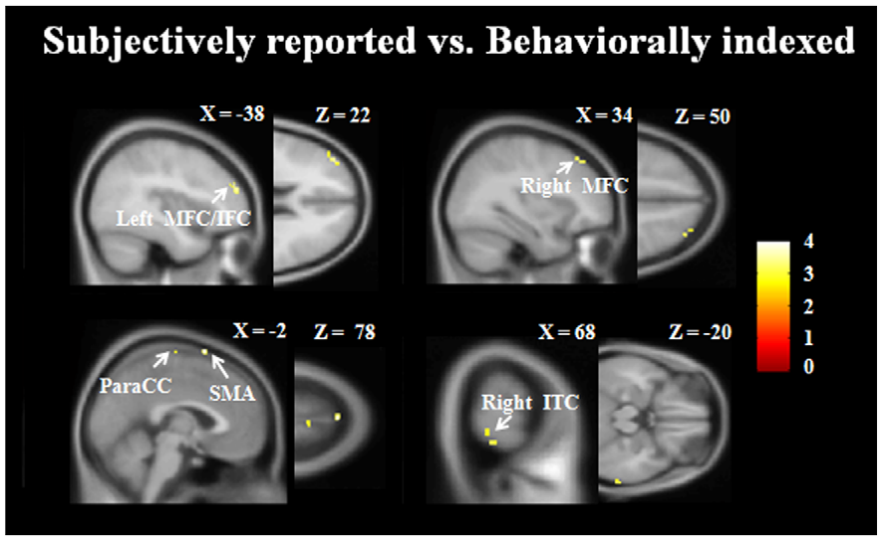
Gamma band beamformer analysis showed increased neural activity associated with subjective reported mind wandering relative to behavioral indexed mind wandering. The results were superimposed on a group-averaged T1 MRI image. X/Y/Z are MNI coordinates. MFC = middle frontal cortex; IFC = inferior frontal cortex; SMA = supplemental motor area; ParaCC = paracentral cortex; ITC = inferior temporal cortex.

We first examined neural activity linked to subjectively reported mind wandering, defined as increased neural activity linked to off-task trials relative to those of on-task trials. We did not observe any significant brain activation within beta and gamma bands under the acceptable threshold (voxels survived an uncorrected *p* value of 0.005, cluster size>10). We then examined neural activities linked to behaviorally indexed mind wandering, defined as increased neural activities linked to missed target trials relative to those linked to hit target trials. There was not any significant brain activation identified within the beta and gamma bands. Given there was significant difference in rhythmic activity between subjectively reported and behaviorally indexed mind wandering, we further examined distinct neural activities between the two by conducting a two-sample t-test on the neural activity linked to these two kinds of mind wandering. Unique to the gamma band and relative to the behaviorally indexed mind wandering, we found that subjectively reported mind wandering showed more activities in the left middle/inferior frontal cortex (x = −38/y = 50/z = 22, *Z* = 3.12; cluster size = 79 voxel), right middle frontal cortex (x = 34/y = 32/z = 50, *Z* = 2.92; cluster size = 14 voxel), supplemental motor area (x = −2/y = 10/z = 78, *Z* = 3.48; cluster size = 17 voxel), paracentral cortex (x = 2/y = −18/z = 76, *Z* = 2.70; cluster size = 11 voxel), and right inferior temporal cortex (x = 68/y = −36/z = −20, *Z* = 3.11; cluster size = 13 voxel); (Fig. 3). Other contrasts failed to show any significant activity.

## Discussion

In the current study, we applied EEG to systematically investigate oscillatory brain activity associated with subjectively reported and behaviorally indexed mind wandering. Subjects more frequently reported feeling unaware of where their attention was focused under off-task conditions, while they were more frequently aware of where their attention was focused under on-task conditions. These results are consistent with previous behavioral studies involving mind wandering and suggest that we often lack awareness that we are off task [3]. In addition, we found the number of off-task trials to be positively correlated with the number of missed target trials. This suggests that a close relationship may exist between subjectively reported and behaviorally indexed mind wandering, and that these two types of mind wandering may share some common mechanisms. Importantly, to the best of our knowledge, our study for the first time shows that increased gamma band activity and activation of frontal-central brain areas are more associated with subjectively reported mind wandering than behaviorally indexed mind wandering.

The difference in gamma band activity between subjectively reported and behaviorally indexed mind wandering supports our hypothesis that executive control processes are more involved in subjectively reported mind wandering. Being that gamma band activity has been considerably linked with attention and memory tasks [14], [19], [20], the difference in gamma band activity observed here may suggest that more attention and memory processes are involved in subjectively reported mind wandering. This falls in line with the characteristics of mind wandering; we attend to our private thought or internal information while becoming directed away from a primary task [3]. The difference could also be reinforced by seemingly distinct neural rhythmic activities associated with these two kinds of mind wandering (these activities were obtained using a liberal threshold: cluster survived a *p* value of 0.05): Subjectively reported mind wandering was additionally associated with increased beta band activity over bilateral frontal and central-parietal areas and decreased alpha band activity over medial central-parietal areas, whereas behaviorally indexed mind wandering was linked to increased alpha band activity over medial frontal areas and increased theta band activity over left frontal-temporal areas. Beta band activity has been suggested to be related to the maintenance of current cognitive states or sensorimotor activities and to contain a strong endogenous top-down component [21]. Enhanced alpha frequency band oscillations have been thought to reflect idling or inhibition of task-irrelevant cortical areas [22], [23]. Theta band activities have been observed in relation to memory retrieval or working memory processing [24], [25], [26]. Thus, the increase of beta and decrease of alpha band activity suggests that subjects maintained an endogenous top-down state during subjectively reported mind wandering; a suggestion that, when considered alongside the increased gamma band activity, supports the involvement of executive control processes. Furthermore, the increase of alpha and theta activity may suggest that less activation of attention networks, but some internal memory retrieval processes, were engaged in behaviorally indexed mind wandering.

After directly comparing the source activity linked to subjectively reported and behaviorally indexed mind wandering, we found that, within the gamma band, subjectively reported mind wandering showed more activation in the bilateral frontal cortex, supplemental motor area, paracentral cortex and right inferior temporal cortex than behaviorally indexed mind wandering. Based upon beamformer analysis, increased bilateral frontal cortex activity was found to be associated with subjectively reported mind wandering. These brain areas have been observed in previous executive control studies [27], [28] and thus support our previously mentioned rhythmic activity results, suggesting that more executive control process are involved in subjectively reported mind wandering. In addition, our results are consistent with a previous fMRI study which used a similar behavioral paradigm [6] and a recent replication of activations in the inferior frontal gyrus during mind wandering [29]. The two types of mind wandering referred to in the present study are both instances of inattention to external information but are characterized with a distinct degree of executive control processing, suggesting that they may exist in different positions along the spectrum of inattention to external activity.

In addition to distinct neural activity linked to subjectively reported and behaviorally indexed mind wandering, beamformer analysis provided additional insight into the neural correlates of mind wandering. Under a liberal threshold (voxels survived an uncorrected *p* value of 0.05, cluster size>100), we found that both subjectively reported and behaviorally indexed mind wandering activated brain areas belonging to the default network, including areas such as medial prefontal cortex and precuneus. These relationships are consistent with previous fMRI studies which have shown mind wandering to be related to default network activation [4], [5], [6]. In addition, under the liberal threshold, we found both subjectively reported and behaviorally indexed mind wandering to be associated with deactivation of bilateral parietal areas belonging to dorsal attention networks [30], [31]. It is reasonable to observe these deactivations to be linked with mind wandering, since deactivation of dorsal attention networks has been found to be a component of the resting state [8], [9], [32]. However, it should be noted that these activities survived under a liberal threshold. They, together with the aforementioned neural rhythmic activities linked to the two kinds of mind wandering (with similar liberal thresholds), should be confirmed with more mind wandering trials in future studies. Additionally, it would also be interesting to determine whether there is any difference in EEG rhythmic patterns between awareness and unawareness of mind wandering (similar to the analysis applied within Christoff et al [6]).

Smallwood and Schooler [3] have suggested that mind wandering recruits executive resources. However, McVay and Kane [33] have proposed that mind wandering represents a failure of executive control. In our study, we showed that the neural activities linked to executive control were involved during mind wandering, seemingly supporting the Smallwood and Schooler's argumentation that mind wandering involves executive function. However, it should be noted that these two points of view are not necessarily conflicting since they merely explain different stages of mind wandering [34]: The control-failure view [33] explains the transient occurrence of mind wandering during demanding tasks, while the executive resources consuming/global availability view explains the mind state after mind wandering has occurred [3], [34]. To fully support such a view, additional studies are needed to systematically examine the entire time course of mind wandering, including its occurrence, duration and disappearance. In addition, we found more executive control processes to be involved in subjectively reported rather than behaviorally indexed mind wandering, suggesting that thought sampling (subjective measurement) may be a better method to effectively measure mind wandering, similar to the idea proposed within consciousness studies [35]. While opposing viewpoints exist with regard to the role of executive processes within mind wandering, they could be used to support the maintenance of internally generated thought (the decoupling hypothesis), or they are geared to suppress the experience of internally generated thought. Although our behavioral data could not differentiate these arguments, activations in both executive related brain areas and the default mode network would support the decoupling hypothesis, which is also evidenced by a recent fMRI study using a similar paradigm [6] and an ERP study involving the processing of irrelevant stimuli during mind wandering [36].

There exists another example of an inattentive state, attention lapse, which could be defined as the failure to detect a readily perceivable target stimulus during a continuous monitoring/vigilance task [37] or a relatively slow response time to identify behaviorally relevant stimuli [38]. Attention lapse was also shown to be linked with an increased activation of the default network in a previous fMRI study [38] and could be predicted by increasing alpha band activity beginning ∼20 s before a missed target [37]. The definition of attention lapse in the previous study [37] is similar to our definition of behaviorally indexed mind wandering, which is evidenced by similar brain activity (increased alpha band activity). Both subjective reports and behavioral errors related to mind wandering have been linked to fluctuations in response time [39], [40]. Thus, subjective mind wandering may share some features with attention lapse, as evidenced by the involvement of default mode activities in the current study. Given that our study indicates subjectively reported mind wandering and behaviorally indexed mind wandering to be distinct in the extent of executive control, it could be suggested that attention lapse may represent a state in which attention is spontaneously shifted away from active tasks, whereas mind wandering is additionally accompanied by the extensive thinking of private thought. However, there is also a possibility that both hit and missed target trials require executive processes, but the contrast process used in the current study canceled out the activations of executive control brain areas (see [41] for further discussion). If this is the case, executive processes would be involved in both subjectively reported and behaviorally indexed mind wandering (attention lapse). Our results might then suggest that behaviorally indexed mind wandering failed to show executive related processes which are related to both types of mind wandering, but with only some default activity.

In conclusion, we observed that, relative to behaviorally indexed mind wandering, subjectively reported mind wandering was associated with increased bilateral frontal-central gamma band activity and corresponding enhanced frontal-central brain activity within the source domain. These dissociate the two types of mind wandering and suggest that a higher degree of executive control processes are involved during subjectively reported mind wandering. These findings indicate the usefulness of subjective measurement in the examination of neurocognitive mechanisms involved in mind wandering, and further imply the necessity of future studies to carefully articulate and manipulate the degree/extent of the inattentive state.

## References

[1] Carrasco M, Eckstein M, Verghese P, Boynton G, Treue S (2009). Visual attention: Neurophysiology, psychophysics and cognitive neuroscience.. Vision Research.

[2] Smallwood J, McSpadden M, Schooler JW (2007). The lights are on but no one's home: Meta-awareness and the decoupling of attention when the mind wanders.. Psychonomic Bulletin and Review.

[3] Smallwood J, Schooler JW (2006). The restless mind.. Psychological Bulletin.

[4] McKiernan KA, D'Angelo BR, Kaufman JN, Binder JR (2006). Interrupting the ‘stream of consciousness’: An fMRI investigation.. Neuroimage.

[5] Mason MF, Norton MI, Van Horn JD, Wegner DM, Grafton ST (2007). Wandering minds: The default network and stimulus-independent thought.. Science.

[6] Christoff K, Gordonb AM, Smallwood J, Smitha R, Schooler JW (2009). Thought samplingduring fMRI reveals default network and executive system contributions to mind wandering.. Proceedings of the National Academy of Sciences of the United States of America.

[7] Smallwood J, Beach E, Schooler JW, Handy TC (2008). Going AWOL in the brain: Mind wandering reduces cortical analysis of external events.. Journal of Cognitive Neuroscience.

[8] Laufs H, Krakow K, Sterzer P, Eger E, Beyerle A (2003). Electroencephalographic signatures of attentional and cognitive default modes in spontaneous brain activity fluctuations at rest.. Proceedings of the National Academy of Sciences of the United States of America.

[9] Mantini D, Perrucci MG, Del Gratta C, Romani GL, Corbetta M (2007). Electrophysiological signatures of resting state networks in the human brain.. Proceedings of the National Academy of Sciences of the United States of America.

[10] Cahn BR, Polich J (2006). Meditation states and traits: EEG, ERP, and neuroimaging studies.. Psychological Bulletin.

[11] Fell J, Axmacher N, Haupt S (2010). From alpha to gamma: Electrophysiological correlates of meditation-related states of consciousness.. Medical Hypotheses.

[12] Scheeringa R, Bastiaansen MC, Petersson KM, Oostenveld R, Norris DG (2008). Frontal theta EEG activity correlates negatively with the default mode network in resting state.. International Journal of Psychophysiology.

[13] Howard MW, Rizzuto DS, Caplan JB, Madsen JR, Lisman J (2003). Gamma oscillations correlate with working memory load in humans.. Cerebral Cortex.

[14] Herrmann CS, Munk MH, Engel AK (2004). Cognitive functions of gamma-band activity: Memory match and utilization.. Trends in Cognitive Science.

[15] Singer JL, Antrobus JS, Sheehan P (1972). Daydreaming, imaginal processes, and personality: A normative study.. The function and nature of imagery.

[16] Delorme A, Makeig S (2004). EEGLAB: an open source toolbox for analysis of single-trial EEG dynamics including independent component analysis.. Journal of Neuroscience Methods.

[17] Morup M, Hansen LK, Arnfred SM (2007). ERPWAVELAB: A toolbox for multi-channel analysis of time-frequency transformed event related potentials.. Journal of Neuroscience Methods.

[18] Maris E, Oostenveld R (2007). Nonparametric statistical testing of EEG- and MEGdata.. Journal of Neuroscience Methods.

[19] Fell J, Fernández G, Peter Klaver P, Elger CE, Fries P (2003). Is synchronized neuronal gamma activity relevant for selective attention?. Brain Research Reviews.

[20] Jensen O, Kaiser J, Lachaux J (2007). Human gamma-frequency oscillations associated with attention and memory.. Trends in Neuroscience.

[21] Engel AK, Fries P (2010). Beta-band oscillations—signalling the status quo?. Current Opinion in Neurobiology.

[22] Klimesch W, Sauseng P, Hanslmayr S (2007). EEG alpha oscillations: The inhibition-timing hypothesis.. Brain Research Reviews.

[23] Palva S, Palva JM (2007). New vistas for α-frequency band oscillations.. Trends in Neuroscience.

[24] Kahana MJ, Seelig D, Madsen JR (2001). Theta returns.. Current Opinion in Neurobiology.

[25] Raghavachari S, Kahana MJ, Rizzuto DS, Caplan JB, Kirschen MP (2001). Gating of human theta oscillations by a working memory task.. Journal of Neuroscience.

[26] Wu X, Chen X, Li Z, Han S, Zhang D (2007). Binding of verbal and spatial information in human working memory involves large-scale neural synchronization at theta frequency.. Neuroimage.

[27] Duncan J, Owen AM (2000). Common regions of the human frontal lobe recruited by diverse cognitive demands.. Trends in Cognitive Science.

[28] Kouneiher F, Charron S, Koechlin E (2009). Motivation and cognitive control in the human prefrontal cortex.. Nature Neuroscience.

[29] Stawarczyk D, Majerus S, Maquet P, D'Argembeau A (2011). Neural correlates of ongoing conscious experience: Both task-unrelatedness and stimulus-independence are related to default network activity.. PLoS One.

[30] Corbetta M, Shulman GL (2002). Control of goal-directed and stimulus-driven attention in the brain.. Nature Reviews Neuroscience.

[31] Fox MD, Corbetta M, Snyder AZ, Vincent JL, Raichle ME (2006). Spontaneous neuronal activity distinguishes human dorsal and ventral attention systems.. Proceedings of the National Academy of Sciences of the United States of America.

[32] Jann K, Kottlow M, Dierks T, Boesch C, Koenig T (2010). Topographic electrophysiological signatures of fMRI resting state networks.. PLoS One.

[33] McVay JC, Kane MJ (2010). Does mind wandering reflect executive function or executive failure? Comment on Smallwood and Schooler (2006) and Watkins (2008).. Psychological Bulletin.

[34] Smallwood J (2010). Why the global availability of mind wandering necessitates resource competition: Reply to McVay and Kane (2010).. Psychological Bulletin.

[35] Lau H, Davies M, Weiskrantz L (2008). Are we studying consciousness yet?. Frontiers of Consciousness: The Chichele Lectures.

[36] Barron E, Riby LM, Greer J, Smallwood J (2011). Absorbed in thought: The effect of mind wandering on the processing of relevant and irrelevant events.. Psychological Science.

[37] O'Connell RG, Dockree PM, Robertson IH, Bellgrove MA, Foxe JJ (2009). Uncovering the neural signature of lapsing attention: Electrophysiological signals predict errors up to 20 s before they occur.. Journal of Neuroscience.

[38] Weissman DH, Roberts KC, Visscher KM, Woldorff MG (2006). The neural bases of momentary lapses in attention.. Nature Neuroscience.

[39] Smallwood J, McSpadden MC, Luus B, Schooler JW (2008). Segmenting the stream of consciousness: The psychological correlates of temporal structures in the time series data of a continuous performance task.. Brain & Cognition.

[40] Smallwood J (2011). The footprints of the wandering mind: Further examination of the time course of an attentional lapse.. Cognitive neuroscience.

[41] Smallwood J, Brown K, Baird B, Schooler JW (in press). Cooperation between the default mode network and the frontal-parietal network in the production of an internal train of thought.. Brain Research.

